# Non-Tumorigenic Pluripotent Reparative Muse Cells Provide a New Therapeutic Approach for Neurologic Diseases

**DOI:** 10.3390/cells10040961

**Published:** 2021-04-20

**Authors:** Toru Yamashita, Yoshihiro Kushida, Koji Abe, Mari Dezawa

**Affiliations:** 1 Department of Neurology, School of Medicine, Dentistry and Pharmaceutical Sciences, Okayama University, Okayama 700-8558, Japan; toruyamashita@okayama-u.ac.jp (T.Y.); abekgabekg@gmail.com (K.A.); 2 Department of Stem Cell Biology and Histology, School of Medicine, Tohoku University, Sendai 980-8575, Japan; y-kushida@med.tohoku.ac.jp

**Keywords:** SSEA-3, pluripotent, MSCs, sphingosine-1-phosphate, ALS, stroke, encephalitis, ischemia

## Abstract

Muse cells are non-tumorigenic endogenous reparative pluripotent cells with high therapeutic potential. They are identified as cells positive for the pluripotent surface marker SSEA-3 in the bone marrow, peripheral blood, and connective tissue. Muse cells also express other pluripotent stem cell markers, are able to differentiate into cells representative of all three germ layers, self-renew from a single cell, and are stress tolerant. They express receptors for sphingosine-1-phosphate (S1P), which is actively produced by damaged cells, allowing circulating cells to selectively home to damaged tissue. Muse cells spontaneously differentiate on-site into multiple tissue-constituent cells with few errors and replace damaged/apoptotic cells with functional cells, thereby contributing to tissue repair. Intravenous injection of exogenous Muse cells to increase the number of circulating Muse cells enhances their reparative activity. Muse cells also have a specific immunomodulatory system, represented by HLA-G expression, allowing them to be directly administered without HLA-matching or immunosuppressant treatment. Owing to these unique characteristics, clinical trials using intravenously administered donor-Muse cells have been conducted for myocardial infarction, stroke, epidermolysis bullosa, spinal cord injury, perinatal hypoxic ischemic encephalopathy, and amyotrophic lateral sclerosis. Muse cells have the potential to break through the limitations of current cell therapies for neurologic diseases, including amyotrophic lateral sclerosis. Muse cells provide a new therapeutic strategy that requires no HLA-matching or immunosuppressant treatment for administering donor-derived cells, no gene introduction or differentiation induction for cell preparation, and no surgery for delivering the cells to patients.

## 1. Introduction

The body infrastructure requires continuous maintenance; body tissues and organs persistently undergo minute damage, which is rapidly and efficiently repaired to maintain tissue homeostasis throughout life. We take this natural reparative activity of the body for granted, and the molecular details of the reparative mechanisms are not yet fully understood.

**Figure 1:**
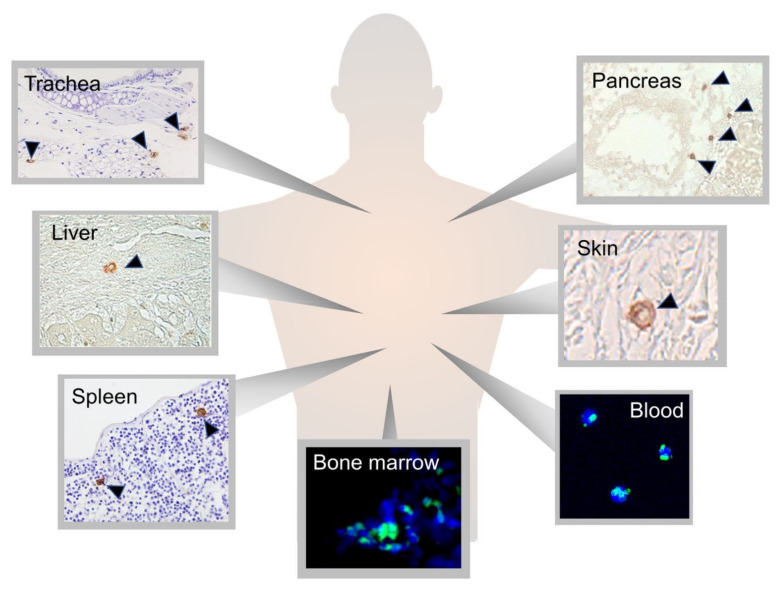
Distribution of Muse cells in the body. Muse cells, detected as SSEA-3(+), distribute in the bone marrow (green signal) [7], peripheral blood (green signal) [4], and connective tissue of various organs, such as the trachea, liver, spleen, pancreas, and skin (brown signal) [8].

**Figure 2:**
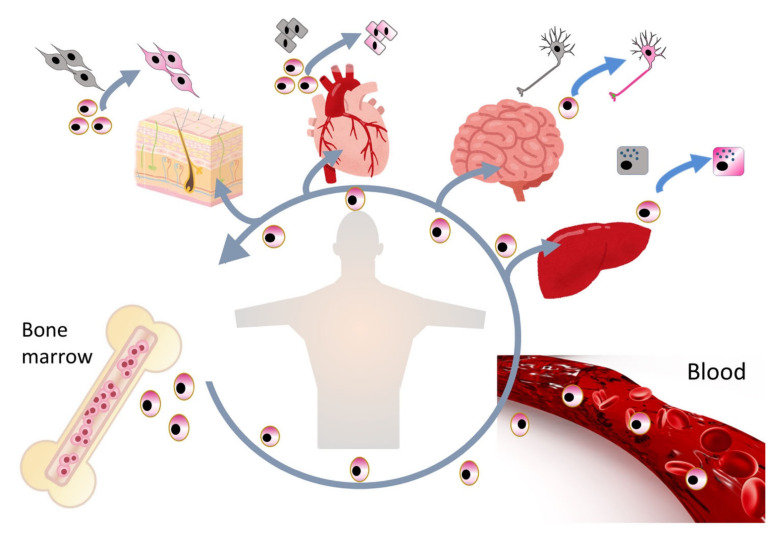
Daily reparative activity of endogenous Muse cells. Muse cells in the bone marrow are considered to be constantly mobilized to the peripheral blood and supplied to every organ, where they replace minutely damaged/apoptotic cells by spontaneous differentiation.

**Figure 3:**
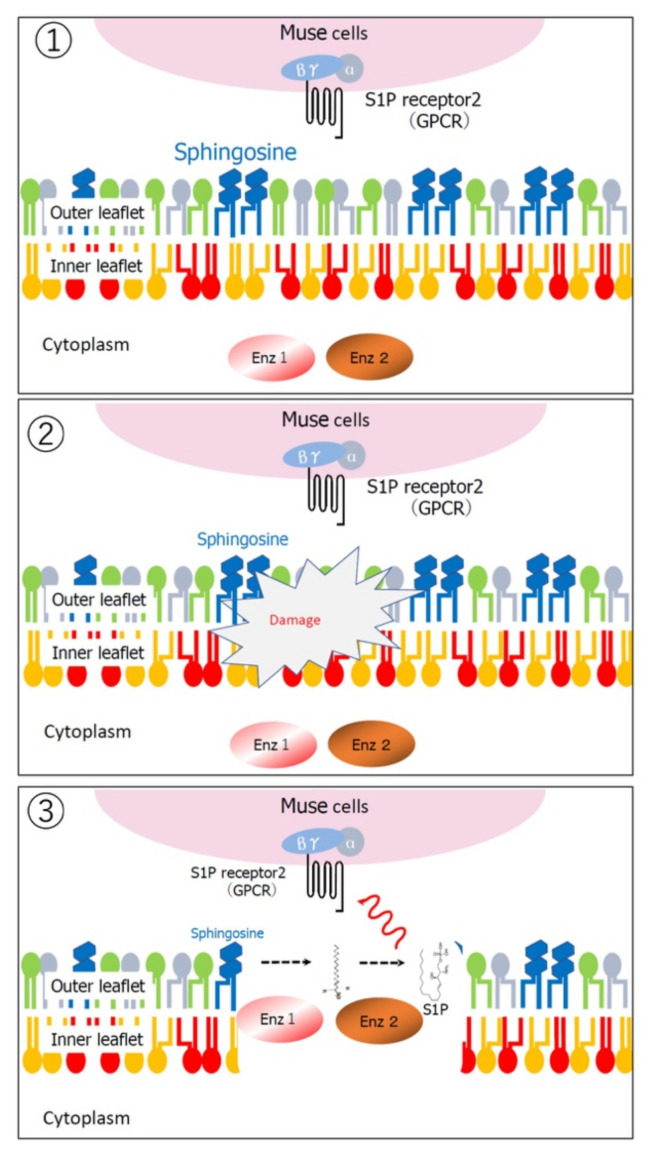
Production of sphingosine-1-phosphate by damaged cells. (**1**) Sphingosine, located in the outer leaflet of the cell membrane, is the substrate of sphingosine-1-phosphate (S1P). (**2**) When the cell membrane is damaged, (**3**) sphingosine reacts with enzymes (Enz1, Enz2) and is phosphorylated to become S1P. Released S1P binds to S1P receptor 2, a G-protein coupled receptor, on Muse cells to attract them to the site of damage. This figure was reproduced with permission from Advances in Experimental Medicine and Biology (Springer, copyright 2018 [2]).

Multilineage-differentiating, stress-enduring (Muse) cells are considered an important part of the body maintenance system. Muse cells, identified as pluripotent surface marker, stage-specific embryonic antigen (SSEA)-3(+), are endogenous, reparative, non-tumorigenic, pluripotent stem cells distributed throughout the body [1,2]. They are constantly mobilized from the bone marrow to the peripheral blood and thus supplied to every organ (Figure 1) [3,4]. Muse cells exhibit pluripotency and are able to differentiate into ectodermal, mesodermal, and endodermal cells and self-renew from a single cell [1]. These beneficial characteristics of Muse cells allow them to differentiate into cells comprising various types of tissue to replenish damaged/lost cells. Tissues comprise a three-dimensional organization of multiple cell types. For tissue repair, Muse cells spontaneously differentiate into multiple cell types comprising the damaged tissue and integrate into the proper position to maintain tissue function [2]. In this manner, they participate in the daily minute repair (Figure 2). Damaged cells actively produce sphingosine-1-phosphate (S1P) by phosphorylating sphingosine, a cell membrane component, and thus, S1P is the general alert signal of tissue damage [5]. Muse cells express S1P receptor 2 (S1PR2), allowing them to sharply sense the S1P alert signal produced by the damaged tissue and selectively home to the site of damage where they accumulate (Figure 3) [6]. 

**Figure 4:**
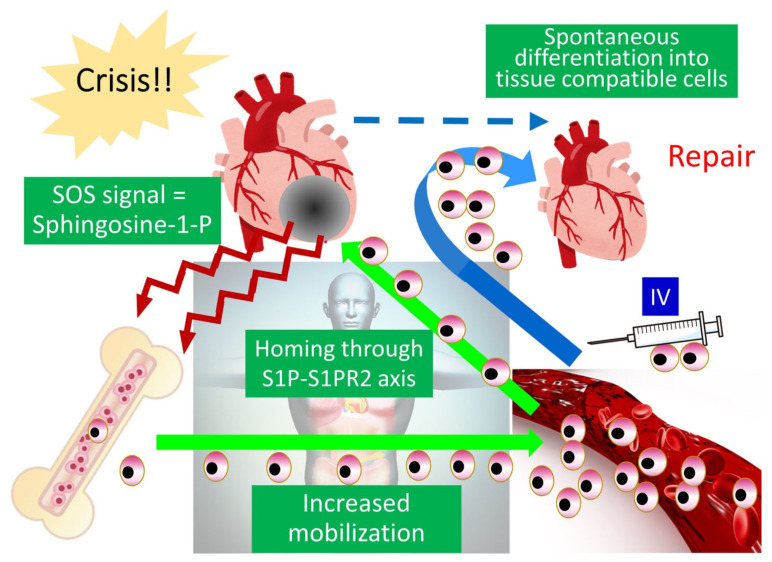
Strategy of Muse cell therapy. For example, in acute myocardial infarction, S1P as an alert signal is produced by the infarcted area and delivered to the bone marrow, where endogenous Muse cells are mobilized to the peripheral blood to increase the number of circulating Muse cells. The circulating Muse cells migrate to the infarcted area via the S1P–S1PR2 axis and replace damaged cells by spontaneous differentiation into tissue-appropriate cells to repair the cardiac tissue. When the number of endogenous Muse cells is insufficient, intravenous administration of exogenous Muse cells enhances the reparative activity, leading to successful tissue repair. This figure was reproduced with permission from Advances in Experimental Medicine and Biology (Springer, copyright 2018 [2]).

Endogenous Muse cells thus act as reparative stem cells through the above-described series of reactions. Suppose that extensive tissue damage is caused, such as by a stroke; the post-infarct tissue produces S1P as a damage alert signal, thereby mobilizing endogenous Muse cells from the bone marrow to the circulating blood to travel to the infarct area and repair the affected tissue. Clinical data support this hypothesis; the number of peripheral blood-Muse cells sharply increases after the onset of stroke and acute myocardial infarction [3,7]. In an acute myocardial infarction study, endogenous Muse cell dynamics in the acute phase was shown to play an important role in the prognosis of patients; patients with a higher number of Muse cells in the peripheral blood in the acute phase exhibited statistically meaningful cardiac function recovery with less occurrence of heart failure at 6 months, compared with another group who did not exhibit an increased number of circulating Muse cell during the acute phase, suggesting their innate reparative function for the heart [3,9]. Thus, the number of endogenous Muse cells is a potential parameter of the body’s reparative activity. If the number of Muse cells is insufficient for repair, or if the patient’s endogenous Muse cells have low reparative activity due to underlying diseases, exogenous Muse cells, collectable from the bone marrow, peripheral blood, organ connective tissues, can be supplied via intravenous infusion to strengthen the body’s reparative activity (Figure 4). This is the basic concept of Muse cell therapy.

Another unique characteristic of Muse cells, compared with other stem cells is their unique immune privilege system. Donor-derived (allogenic) Muse cells do not require human leucocyte antigen (HLA)-matching or long-term immunosuppressant treatment [6]. Thus, on the basis of these characteristics, Muse cells provide simple, sophisticated next-generation medical care that can be implemented not only in advanced medical institutions but also in general hospitals and clinics due to the following advantages:

- Muse cells are endogenous and therefore elicit few safety concerns.
- Muse cells can be delivered intravenously and do not require surgery for their administration.
- Muse cells do not require gene introduction or cytokine treatment to be rendered pluripotent and induce differentiation.
- Donor Muse cells can be used without HLA-matching or immunosuppressant treatment.
- Muse cells remain incorporated as functional cells in the host tissue for an extended period of time, making their anti-inflammatory, anti-apoptotic, and trophic effects long-lasting.

**Figure 5:**
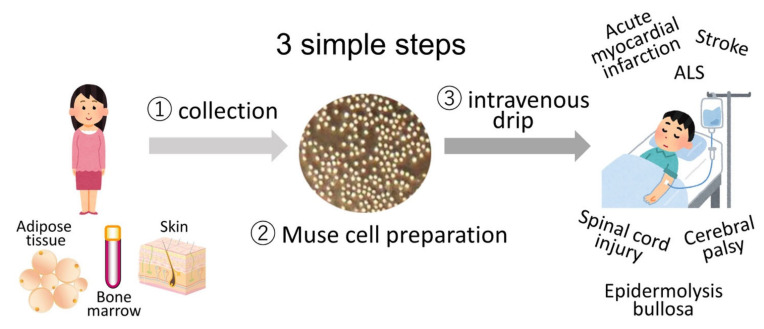
Strategy for Muse cell clinical trials consists of 3 simple steps. Muse cells, collectable from donor sources such as the bone marrow, adipose tissue, and skin, are expanded to produce Muse cell preparations and directly delivered to patients with acute myocardial infarction, stroke, epidermolysis bullosa, spinal cord injury, cerebral palsy, and ALS by intravenous drip without HLA-matching and immunosuppressant treatment.

Clinical trials in which donor Muse cells are delivered by intravenous infusion without HLA-matching and immunosuppressant treatment to patients with acute myocardial infarction [10], stroke, epidermolysis bullosa [11], spinal cord injury, neonatal cerebral palsy, and amyotrophic lateral sclerosis (ALS) are in progress (Figure 5). 

In this review, we discuss the potential therapeutic application of Muse cells for ALS and other neurologic diseases.

## 2. Basic Characteristics of Muse Cells

### 2.1. Muse Cells as Endogenous Reparative Stem Cells Are Widely Distributed in the Body

Muse cells are identified as cells positive for stage-specific embryonic antigen (SSEA)-3, a representative marker of pluripotent stem cells [1]. SSEA-3 is an antibody that recognizes a sugar epitope on the cell surface of pluripotent/totipotent cells, such as embryonic stem (ES) cells and epiblast stem cells in the two-cell stage during normal development [12,13]. Because sugar is the epitope recognized by the SSEA-3 antibody, there is no gene that directly encodes SSEA-3; thus, specific knockout animals for SSEA-3 are difficult to generate. On the other hand, species differences in SSEA-3 do not exist, and SSEA-3 can thus be applied to identify Muse cells across species. In fact, Muse cells have been identified in several mammals, including mouse, rat, rabbit, goat, and swine, using SSEA-3 [6,14,15,16,17]. 

Muse cells widely distribute throughout the body; they are found in the bone marrow, peripheral blood, and connective tissue of nearly every organ (Figure 1) [8,18]. They are also found in extra-embryonic tissues such as the umbilical cord, which is rich in connective tissue [19]. Muse cells comprise approximately ~0.03% (1:3000) of mononucleated cells in the bone marrow and form loose clusters near blood vessels [1,7]. They are constantly mobilized from the bone marrow to the peripheral blood at a rate of approximately 0.01~0.2% of the mononucleated cell fraction in the blood. Large individual differences exist, however, and the rate varies greatly even within individuals, depending on the physical condition and presence of illness or injury [3,4]. 

Muse cells positive for SSEA-3 can be recognized in connective tissue. Somatic stem cells are known to have their own niche, an area of a tissue that provides a specific microenvironment in which stem cells maintain a quiescent state, e.g., hair follicle and hematopoietic stem-cell niches [20,21]. To date, Muse cells have not been observed in any particular niche-like tissue structures. Rather, they are freely and sparsely distributed in the connective tissue, probably due to their continuous active dynamic movement in vivo [18]. 

Besides SSEA-3, Muse cells express the pluripotent master genes Oct3/4, Nanog, and Sox2, as well as other pluripotency-related markers, such as Rex1, PAR4, BMP4, CBX7, DAZL, DPPA3, DPPA4, FGFR1, GDF3, KLF4, MSX2, Myc, NR0B1, Prdm1, Six4, SPRY1/2, SSBP2, and UTF1 [18]. A single Muse cell can generate cells representative of all three germ layers; in suspension culture, single Muse cells form embryoid-like clusters [1]. The cluster cells spontaneously generate endodermal (positive for GATA-6, cytokeratin 7, and alpha-fetoprotein), mesodermal (positive for Nkx2.5, smooth muscle actin, and desmin), and ectodermal (positive for MAP-2 and neurofilament) cells without any cytokine induction when transferred onto gelatin-coated culture dishes for expansion [1]. Such triploblastic differentiation from a single cell can be reproduced over generations, indicating self-renewability. In addition to their spontaneous differentiation, Muse cells also differentiate in vitro at a high rate (~80–95%) into various target cell types, such as hepatic-, cardiac- and neural-lineage cells, as well as into adipocytes, osteocytes, keratinocytes, and melanocytes, when certain sets of cytokines are supplied in a step-wise manner [22,23,24]. Because of these two core characteristics, triploblastic differentiation and self-renewability at a single cell level, Muse cells are considered pluripotent stem cells.

### 2.2. Sources of Muse Cells

Muse cells are collectable as SSEA-3(+) cells from various sources, such as the bone marrow, peripheral blood, and connective tissue of nearly every organ, including extraembryonic tissues, such as the umbilical cord [1,18,19]. Importantly, commercially available cultured mesenchymal stem cells (MSCs) established from bone marrow, adipose tissue, and umbilical cord, as well as from dermal fibroblasts, are also practical sources of Muse cells [25]. Several percent of the total population of MSCs and fibroblasts can be collected as SSEA-3(+) Muse cells [22]. 

The core pluripotent characteristics, triploblastic differentiation and self-renewability at a single cell level, are consistently exhibited by Muse cells derived from different sources. Muse cells exhibit their own differentiation directivity, however, according to their source. Adipose tissue-derived Muse cells express higher levels of genes related to their differentiation into adipocytes, osteocytes, and skeletal muscle cells than bone marrow- and dermal fibroblast-derived Muse cells [26]. Bone marrow-derived Muse cells, however, contain the highest levels of factors related to hepatocytes and pancreatic cells. Both bone marrow- and dermal fibroblast-derived Muse cells exhibit higher expression levels of neuronal-, melanocyte-, and epidermal-related genes, compared with adipose tissue-derived Muse cells [26]. Thus, the differentiation propensity is not exactly the same among Muse cells obtained from different sources. 

Peripheral blood-derived Muse cells are unique in that, while they exhibit pluripotency gene expression, triploblastic differentiation, and migration toward S1P, like Muse cells derived from other sources, they consistently express the surface marker CD45, a general white blood cell marker, along with SSEA-3, unlike other Muse cells [4]. CD45 expression has never been observed in bone marrow-, dermal fibroblast, and adipose tissue-derived Muse cells, and this is a unique feature of peripheral blood-derived Muse cells. In addition, the gene expression levels of Oct3/4, Nanog, and Sox2 are higher than that in other Muse cells [4]. Generally, in suspension, Muse cells have substantially increased Oct3/4, Nanog, and Sox2 gene expression levels, compared with those in an adherent state [27]. Muse cells in the peripheral blood are always in suspension, which might be the reason for their higher pluripotency gene expression levels. 

### 2.3. Stress Tolerance, High DNA Repair Ability, and Non-Tumorigenicity 

Muse cells secrete a number of factors related to stress tolerance, such as 14-3-3 protein, which plays a role in DNA repair and apoptosis inhibition [28]. The ability to repair DNA is necessary to maintain genome integrity and normal function, particularly in stem cells. Muse cells have a higher capacity for DNA repair, compared with other somatic stem cells such as MSCs [29]. As mentioned above, Muse cells comprise several percent of cultured MSCs. When MSCs are separated into Muse and non-Muse [SSEA-3(-)]-MSCs and compared after ultraviolet light or H_2_O_2_ exposure, Muse cells exhibit lower rates of apoptosis and senescence than the non-Muse MSCs. This is partly explained by the high expression levels of ataxia-telangiectasia mutated kinase and γ-H2AX, which are relevant to DNA repair, and of enzymes related to non-homologous end-joining [29]. Importantly, a higher capacity for DNA repair contributes to the low risk of tumorigenesis of Muse cells and makes them more resistant to the accumulation of mutations. 

Although Muse cells exhibit pluripotency, they have low telomerase activity, comparable to that of somatic cells, and do not form teratomas for up to 6 months when transplanted into the testis of immunodeficient mice [22,26,30]. In fact, gene expression levels of factors relevant to cell cycle progression in Muse cells are similar to those in somatic cells and substantially lower than those in ES and induced pluripotent stem (iPS) cells [22]. On the other hand, the proliferation speed of Muse cells is ~1.3 days/cell division, similar to or slightly slower than that of fibroblasts, and they are thus expandable to clinical scale [1]. Thus, Muse cells are pluripotent, endogenous, and non-tumorigenic. 

### 2.4. Ability to Selectively Home to Sites of Damage

The S1P–S1PR2 system is the main axis that controls the selective homing of circulating Muse cells, either endogenous or exogenously administered, to sites of damage [6]. This feature was confirmed by S1PR2 knockdown with small interference RNA in Muse cells or co-administration of the S1PR2-specific antagonist JTE-013 in Muse cells. In both cases, the specific homing of Muse cells to the sites of damage was largely impeded. On the other hand, the S1PR2 agonist SID46371153 strongly enhances the migration of Muse cells in vitro [6]. There are five S1PR subtypes, S1PR1 through S1PR5. Muse cells express all five subtypes, but the expression of S1PR2 is highest among the five subtypes [6]. 

**Figure 6:**
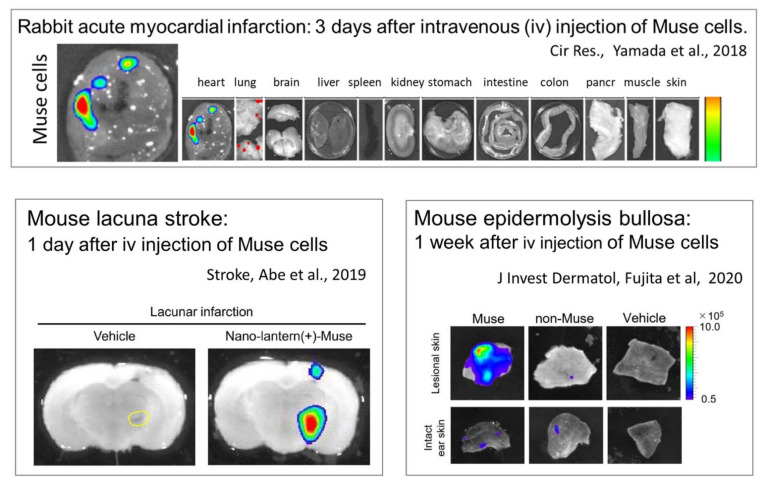
In vivo dynamics of intravenously injected exogenous Muse cells in animal models. Nano-lantern-labeled human Muse cells were intravenously injected into a rabbit model of acute myocardial infarction (3 days after), a mouse model of lacunar infarction (1 day after), and a mouse model of epidermolysis bullosa (1 week after). Muse cells selectively accumulated in the damaged tissue in all models.

S1P is a sphingolipid produced from sphingosine, a component of the outer leaflet of the cell membrane, by converting enzymes SPHK1 and SPHK2 [31]. The synthesis of S1P in cells is activated by a variety of stimuli, including tissue damage and inflammation (Figure 3) [5]. Notably, the S1P–S1PR2 axis by which Muse cells selectively home to sites of damage is active regardless of the tissue or organ type (Figure 6). Intravenously injected MSCs are mostly trapped in the lung capillaries, and it is now known that the main benefit of MSCs is their trophic effect, rather than cell replacement by differentiation [32]. In a rabbit acute myocardial infarction model, ~14.5% of intravenously injected Muse cells engrafted into the post-infarct heart at 3 days, whereas only a few or no MSCs integrated into the heart in the same model [6] (Figure 6). In mouse models of epidermolysis bullosa [33], lacunar stroke l [34], doxorubicin-induced nephropathy [35], ALS [36], and Shiga toxin-producing *Escherichia coli* (STEC)-associated encephalopathy [37], as well as in rat models of middle cerebral artery occlusion ischemia and perinatal hypoxic ischemic encephalopathy [38,39], Muse cells exhibited superiority over MSCs/non-Muse MSCs in selective homing to the sites of damage. Not only in these animal models, but also the data collected from patients with stroke and acute myocardial infarction demonstrated that an increase in the serum S1P level precedes the increase in the number of circulating endogenous Muse cells after the cell injury onset [3,7]. These findings indicate that the S1P–S1PR2 axis is the key system that controls the selective homing of circulating Muse cells to sites of damage. 

Other than the S1P–S1PR2 axis, Muse cells also rely on the SDF-1-CXCR4 and HGF-c-Met systems, which are the main axes of MSC homing, but to a lesser extent, compared with the S1P–S1PR2 axis, because blocking these systems only partially suppresses the selective migration of Muse cells to damaged tissue [27].

### 2.5. Replacement of Damaged/Apoptotic Cells by Spontaneous Differentiation of Muse Cells into the Damaged/Apoptotic Cell Type

**Figure 7:**
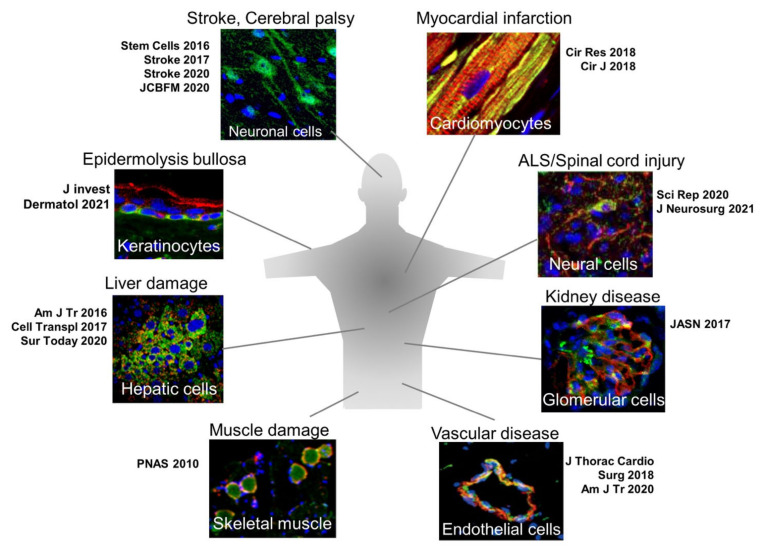
Spontaneous differentiation of homed Muse cells in each damaged tissue. Muse cells after homing to the damaged tissue spontaneously differentiate into tissue-comprising cells.

Muse cells are able to differentiate into a variety of cells of triploblastic-lineages both in vitro and in vivo [18]. An outstanding characteristic of Muse cells that is most relevant to their reparative functions is their ability to spontaneously and simultaneously differentiate with few errors into the damaged/apoptotic cell types that comprise the tissue to which Muse cells homed via the S1P–S1PR2 axis (Figure 7). 

Another characteristic is that in vivo differentiation proceeds rapidly, compared with in vitro cytokine-induced differentiation. When proper sets of cytokines are supplied in vitro, more than 80% of Muse cells differentiate into melanocytes, cardiomyocytes, osteocytes, adipocytes, and neural- and hepatic-lineage cells, but generally it takes at least several months [22,23,24]. In vivo, however, Muse cells that homed to the post-infarct area of a stroke model spontaneously differentiated into neuronal cells (~60% of engrafted cells) and oligodendrocytes (12~25%) with a rapid time course: elongated neurites and expressed progenitor markers NeuroD and Mash1 within 3 days, forming a network-like structure, and expressed maturity markers MAP2 and NeuN at 7 days [34,38]. Muse cell-derived neuronal cells incorporate into the pyramidal tract, including the pyramidal decussation, as demonstrated by anterograde and retrograde tracing, and into the sensory tract, as demonstrated by somatosensory-evoked potentials and the formation of synapses with host neuronal cells at 3 months, leading to statistically meaningful functional recovery [34,38]. Spontaneous differentiation of Muse cells into neuronal and glial cells after homing to the damaged central nervous system is also reported in other models, perinatal hypoxic ischemic encephalopathy, brain hemorrhage, ALS, and STEC-related encephalopathy [36,37,39,40]. In an acute myocardial infarction model, Muse cells homed to the post-infarct tissue and within 2 weeks spontaneously differentiated into cells positive for cardiomyocyte markers, such as troponin-I, sarcomeric α-actinin, and connexin 43, exhibiting calcium influx and efflux synchronous with heart activity recorded by an electrocardiogram [6].

In mouse liver damage models, human Muse cells expressed CK19, DLK, OV6, and alpha-fetoprotein, markers of liver progenitor cells, at 2 days after intravenous injection and expressed mature hepatocyte markers HepPar1, albumin, and anti-trypsin within 2 weeks [27,41]. Muse cell-derived hepatocytes that did not fuse with host hepatocytes in fluorescence in situ hybridization expressed cytochrome P450, family 1, subfamily A, polypeptide2, and glucose-6-phosphatase, enzymes related to drug metabolism and glycolysis, and delivered increased serum albumin and decreased total bilirubin levels, suggesting that the Muse cells were functioning as hepatocytes [27,41]. The same tendency was reproduced in a swine hepatectomy model intravenously injected with allogenic-Muse cells [16].

In a mouse chronic kidney disease model, intravenously injected human Muse cells homed to the site of damage and spontaneously differentiated into podocytes (positive for WT-1 and podocin), mesangial cells (positive for megsin), and endothelial cells (positive for CD31 and von Willebrand factor), which are components of the glomerulus, without fusion and improved creatinine clearance, urine protein, and plasma creatinine [35]. Human Muse cells differentiated spontaneously into dystrophin(+) skeletal muscle cells in a mouse muscle degeneration model [1]; into endothelial cells and smooth muscle cells in mouse aortic aneurism model [42]; and into keratinocytes, hair follicular cells, sweat gland cells, and capillary cells in an mouse epidermolysis bullosa model [33]. 

In the above-mentioned reports, Muse cells were not pretreated with cytokines or gene introduction for differentiation into purposive cells prior to administration, which is required for general ES/iPS cell transplantation. The rapid progression of in vivo differentiation of Muse cells sharply contrasts with the in vitro differentiation of Muse cells and of ES/iPS cells, which require at least several weeks to several months of induction procedures to generate mature differentiated cells. Therefore, the mechanism of in vivo differentiation in Muse cells is presumed to substantially differ from those pluripotent stem cells. 

To investigate whether Muse cells differentiate by fusing with resident cells, fluorescence in situ hybridization (FISH) was performed in several animal models. In a mouse liver fibrosis model in which hepatocyte fusion occurs naturally, 2.6 ± 0.2% of HepPar-1(+)-human Muse cells incorporated into the liver tissue were suggested to fuse with host mouse hepatocytes [27]. On the other hand, in a rabbit acute myocardial infarction model, the majority of GFP(+)/sarcomeric α-actinin(+) human Muse cells that were incorporated into post-infarct regions reacted to the human-FISH probe but not to the rabbit probe, and only 0.33 ± 0.06% of the cells were positive for both the human and rabbit probes [6]. This finding was further supported by qPCR of the human-specific Alu sequence; 7 pg of the Alu sequence was detected per nanogram of rabbit heart tissue DNA, confirming the integration of human Muse cells into the rabbit heart [6]. Similarly, in a mouse chronic kidney disease model given an intravenous injection of human GFP(+)-Muse cells, no human/mouse probe double-positive cells among the GFP(+)/WT1(+) cells were observed in the glomerulus [35]. Together, these results suggest that fusion between Muse cells and host cells is not likely a major mechanism of Muse cell differentiation in damaged tissue.

### 2.6. Immune Privilege of Muse Cells

Immunologic rejection is a main drawback of allogeneic-based cell therapy [43]. Based on their immunomodulatory effects, MSCs are applied for graft-versus-host disease therapy in some countries [44,45]. Even for allogenic MSCs, however, immunologic rejection has been reported [44].

Muse cells possess unique immunomodulatory properties: allogenic Muse cells can survive and are incorporated into rabbit acute myocardial infarction host tissue for an extended period (>6 months), even without immunosuppressant treatment [6]. In both normal (Wistar) rats and immunocompromised (SCID) mice, intravenously injected human Muse cells survive as neuronal and glial cells in the ischemic brain tissue for 6 months, while MSCs or cells other than Muse cells in MSCs (i.e., SSEA-3(-) non-Muse MSCs that correspond to ~98% of total MSCs) became undetectable in all the tissues in the body within 2 weeks [34,39]. Interestingly, these species-mismatch experiments were conducted without immunosuppressant treatment. Even in non-immunocompromised mice, such as BALB/c mice, intravenously injected human Muse cells homed to damaged glomeruli and survived as glomerular cells for nearly 2 months without immunosuppressant treatment, while non-Muse MSCs became undetectable in the body within 2 weeks [35]. 

These observations demonstrate that Muse cells have higher immunomodulation ability than general MSCs and non-Muse MSCs. Muse cells express HLA-A, -B, and -C (MHC class I), but not HLA-DR (MHC class II) on the cell surface. They also express a special class of HLA molecules, HLA-G [6]. HLA-G, first discovered in immune-privileged extravillous trophoblasts, can strongly suppress the immune response or inhibit the proliferation and maturation of macrophages, T and B cells, NK cells, dendritic cells, and neutrophils [46]. Therefore, HLA-G expression is suggested to protect Muse cells from immunologic attack after intravenous injection.

When cocultured with naïve T cells, Muse cells can induce naïve T cells to differentiate into regulatory T cells. Muse cells can also suppress dendritic cell differentiation in vitro [6]. The immunomodulation can be explained by the production of indoleamine-2,3 dioxygenase in Muse cells, which is an immunosuppressive factor [35]. 

Because of long-lasting immunomodulatory effects, neither immunosuppressant treatment nor HLA-matching test before administration are necessary for the application of donor-Muse cells. 

### 2.7. Bystander Effects of Muse Cells on Tissue Repair

Because Muse cells remain in the host tissue as functional cells for an extended period of time, the anti-inflammatory, anti-apoptotic, and trophic effects brought by Muse cells are long-lasting and effective. Muse cells secrete a variety of factors, including PDGF-A, PDGF-BB, EGF, HGF, VEGF, IL-6, KGF, PGE2, ANG1, TGF-β, bFGF, and SDF-1 that promote wound healing and inhibit apoptosis [6,17,35,47,48]. Hepatocyte growth factor (HGF) and vascular endothelial growth factor (VEGF), which are protective against general tissue damage, are suggested to promote tissue repair in acute myocardial infarction and liver damage models treated with Muse cells [6,48]. In a rat extra-small partial liver transplantation model, HGF and VEGF, which are more highly expressed in Muse cells than in non-Muse MSCs, had a statistically meaningful protective effect on liver sinusoidal endothelial cells [48]. In addition, in a swine hepatectomy model, animals that received allogenic Muse cells exhibited less necrosis, compared with animals that received allogenic MSCs [16]. In damaged brain tissue, Muse cells ameliorated the effects of excitotoxic brain glutamatergic metabolites and suppressed microglial activation, as shown by magnetic resonance spectroscopy and positron tomography, respectively [39]. Thus, Muse cells have greater tissue protective effects than MSCs.

With regard to their anti-inflammatory effect, Muse cells actively produce interleukin-10, transforming growth factor-β, and prostaglandin E2 [17,47]. Granulocyte colony stimulating factor production was recently demonstrated to play a central role in the activities of Muse cells to protect the blood–brain barrier and neural cells in STEC-associated encephalopathy [37].

Muse cells can also produce matrix metalloproteases-1 (MMP1), MMP2, and MMP9. Notably, MMP9 is only produced by Muse cells and not by non-Muse MSCs [6]. MMPs are important for suppressing fibrosis, because they degrade the extracellular matrix. In studies performed using animal models of liver damage, chronic kidney disease, and acute myocardial infarction, intravenous injection of Muse cells provided a statistically meaningful reduction of fibrosis, compared with the MSC/non-Muse MSCs and vehicle groups [9,16,27,40,49].

As mentioned above, Muse cells produce factors that promote neovascularization, represented by VEGF and HGF [6,48]. Several reports, however, indicate that infused Muse cells directly participate in neovascularization by spontaneously differentiating into vascular cells after homing to damaged tissues, such as the post-infarct heart, damaged glomeruli, and damaged liver [6,35,48]. More directly, intravenously injected human Muse cells differentiated into vascular components, CD31+ endothelial cells in the intimal layer and smooth muscle cells in medial layer in a mouse aortic aneurism model [42]. Thus, Muse cells are efficient in vascular protection, as well as in neovascularization.

## 3. Comparison of the Reparative Effects of Muse Cells and MSCs

The outcomes of tissue repair, functional recovery, anti-apoptotic, anti-inflammatory, and anti-fibrosis effects are all consistently superior in animals injected with Muse cells (Muse group), compared to animals injected with MSC/non-Muse MSCs (MSC/non-Muse MSC group) in disease models. In a rabbit acute myocardial infarction model, for example, the infarct size reduction was ~2.5-fold greater in the Muse group than in the MSC/non-Muse MSC group both at 2 weeks and 2 months [6]. Functional recovery in rat/mouse stroke and hypoxic ischemic encephalopathy models also exhibited statistically meaningful superiority of the Muse group over the MSC/non-Muse MSC group (*p* < 0.001 and *p* < 0.01) in the modified neurologic severity, rotarod, and other neurologic function scores at 3 months or even beyond 3 months after administration [38,39]. Statistically significant anti-apoptotic, anti-inflammatory, and anti-fibrosis effects were observed in the Muse cell group, compared with the MSC/non-Muse MSC group in models of mouse chronic kidney disease, mouse hepatitis, and rat lung ischemic-reperfusion [27,35,47]. These differences between Muse cells and MSCs are considered to arise from the differences in their specific homing abilities, the length of survival in the host tissue after intravenous injection, differentiation potential, and immunomodulation. Unlike Muse cells, MSCs do not home to damaged tissue, nor do they remain in the tissue or the body for more than 2 weeks after administration [6,27,35,37,39]. MSCs differentiate into osteocytes, adipocytes, and chondrocytes with lower efficiency than Muse cells and are unable to differentiate into other mesodermal cells or into ectodermal or endodermal lineage cells [26]. 

### Comparison with Other Stem Cells

Muse cells are distinct from other pluripotent stem cells, such as ES/iPS cells [22], as well as from other somatic stem cells claimed to be pluripotent, such as very small embryonic-like (VSEL) stem cells [50] and multipotent adult progenitor cells (MAPCs) [51], in terms of their proliferative activity, morphology, marker expression, and tissue distribution. Muse cells express pluripotent markers, as mentioned above, and exhibit triploblastic differentiation ability and self-renewability at the single cell level. Compared with ES/iPS cells, however, Muse cells exhibit moderate pluripotency gene expression and are non-tumorigenic; Nanog, Sox2, and Oct3/4 expression levels are lower in Muse cells than in ES/iPSCs, but higher in Muse cells than in general somatic cells, such as fibroblasts [22]. Observations regarding the methylation of the Nanog and Oct3/4 promoter regions support this; these promotor regions are less methylated in Muse cells than in general fibroblasts, whereas those in iPS cells are fully demethylated [18]. On the other hand, expression of genes relevant to the cell cycle is lower in Muse cells than in ES/iPS cells, consistent with the fact that Muse cells exhibit pluripotency but are non-tumorigenic [22]. In relation with this, telomerase activity, an indicator of tumorigenic proliferation, is the same between Muse cells and somatic cells but substantially lower in Muse cells than in iPSCs [22]. The proliferation speed of Muse cells is ~1.3 days/cell division, and expansion is stable until they reach the Hayflick limit; thus, while growth continues on a clinically relevant scale, Muse cells do not proliferate exponentially, unlike ES/iPS cells [1]. Indeed, Muse cells transplanted into the testes of immunodeficient mice did not generate any tumors for up to 6 months [22,30]. 

Pluripotent stem cells were recently classified into two discrete states, ‘naïve’ and ‘primed’, based on their growth factor dependency, intracellular signaling, marker expression, and differentiation potential [52]. For example, naïve pluripotent stem cells depend on the LIF/STAT3 and BMP4 pathways or LIF + small molecule inhibitors (called 2i) to sustain their self-renewability [53], whereas primed pluripotent stem cells depend mainly on FGF and activin/transforming growth factor β to stably promote their self-renewal [54]. Muse cell properties are more similar to those of primed pluripotent stem cells than naïve pluripotent stem cells, such as ES/iPS cells, because Muse cells do not require FGF to maintain their proliferation and self-renewal abilities. 

VSELs (3–5 μm; smaller than red blood cells) found in the peripheral blood, umbilical cord blood, and reproductive tissues exhibit positivity for Sca1, CD34, CXCR4, and SSEA-1 and negativity for Lin and CD45 [50]. In contrast to VSELs, Muse cells are found not only in the bone marrow and peripheral blood but also in organ connective tissue. Human Muse cells in the bone marrow and organs are 13~15 μm, and those in the peripheral blood are ~10 μm; thus, Muse cells are considerably larger than VSELs [1,4]. Marker expression also differs between Muse cells and VSELs; Muse cells from the bone marrow and organs are double-positive for SSEA-3 and CD105, an MSC marker, whereas peripheral blood-derived Muse cells are consistently double-positive for SSEA-3 and CD45, a white blood cell marker. The expression of CD45 differs between Muse cells and VSELs. In addition, VSELs are positive for CD34, whereas Muse cells are not [1,4]. 

MAPCs (8~10 μm), which locate in the bone marrow, are positive for markers related to MSCs (CD13, CD44, CD73, CD90, and CD105) and negative for hematopoietic (CD34, CD45, and CD117) and endothelial (CD34 and CD309) markers [51]. Bone marrow- and organ-derived Muse cells express MSC markers, such as CD29, CD90, and CD105, together with SSEA-3 [22], whereas peripheral blood-Muse cells express CD45 and SSEA-3 [4]. 

Together, these findings clearly demonstrate that Muse cells are distinct from ES/iPS cells, VSELs, and MAPCs.

In the following sections, we review the results of Muse cell-based preclinical studies of ALS, as well as other neurologic diseases.

## 4. Muse Cell-Based Preclinical Studies in Neural Diseases

### 4.1. ALS

ALS is a fatal neurodegenerative disease that usually develops after middle age. Motor neurons selectively degenerate, resulting in limb weakness and muscle atrophy, dysarthria, and swallowing disorders. Respiratory failure due to respiratory muscle paralysis progresses, and ALS patients usually die within 3 to 5 years after onset [55]. 

**Figure 8:**
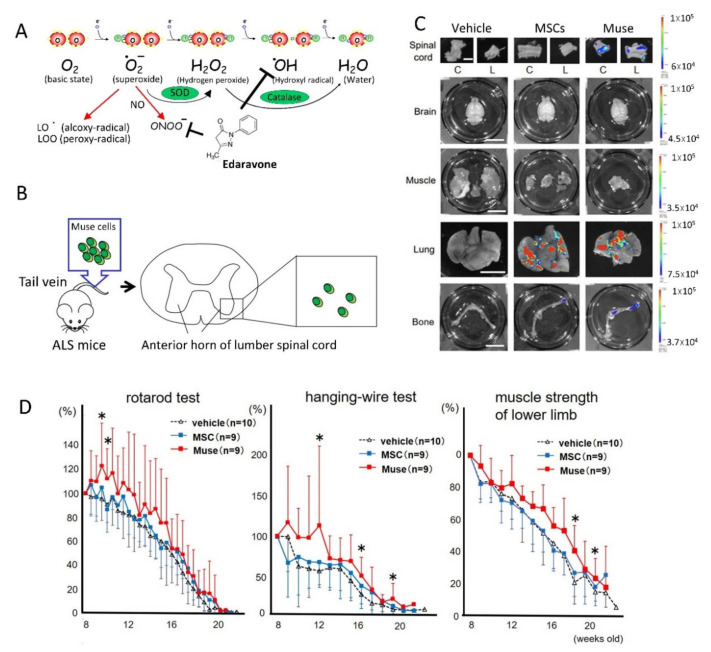
Effectiveness of human Muse cells in a mouse ALS model. (**A**) Free radicals and mechanism of action of a free radical scavenger, edaravone. Free radicals, such as hydroxyl radicals (^•^OH), are produced under pathologic conditions. Hydroxyl radicals are extremely reactive among reactive oxygen species and oxidize biologic components, such as proteins, lipids, sugars, and nucleic acids, causing neuronal cell death. On the other hand, edaravone is thought to mainly detoxify hydroxyl radicals by donating electrons. (**B**) Schema of intravenous administration therapy with Muse cells for ALS mice. Muse cells administered by tail vein injection migrated to the lumbar spinal cord and survived for a long period of time. (**C**) In vivo dynamics of Nano-lantern-labeled MSCs and Muse cells. Only Muse cells and not MSCs homed to the cervical (C) and lumbar (L) spinal cord. Both MSCs and Muse cells were detected in the lung and the femur. (**D**) Clinical analysis of ALS mice treated with vehicle, MSC, and Muse cells (modified from Yamashita et al., 2020 [36]). Mice receiving Muse cells showed significant improvement in the rotarod test, hanging-wire test, and lower limb muscle strength (* *p* < 0.05 vs. vehicle). The figure is reproduced from Yamashita et al., Sci Rep, 2020 [36].

ALS is classified into sporadic ALS and familial ALS, which are associated with mutations in Cu/Zn superoxide dismutase (SOD1) [56,57], TAR DNA binding protein 43 (TDP-43) [58], and a hexanucleotide repeat expansion of the C9orf72 gene [59]. Interestingly, SOD1 is a key enzyme that detoxifies the free radical superoxide, indicating that free radicals play an important role in the pathophysiology of familial ALS (Figure 8A). Involvement of the abnormally folded SOD1 protein is also reported in the pathophysiology of sporadic ALS, and it is presumed that free radical damage due to the abnormal SOD1 protein may be involved in the pathology of sporadic ALS [60]. The autopsied brains of ALS patients have decreased expression of an active oxygen detoxifying enzyme (glutathione peroxidase), increased expression of a DNA peroxidation index (8-OHdG), and increased expression of lipid peroxidation markers [61]. In a study using SOD1^G93A^ transgenic mice (ALS model mice), 8-OHdG expression was observed even before the appearance of clinical symptoms [62]. Recent studies revealed that MMP activation associated with neurovascular unit damage occurs in the anterior horn of the spinal cord of ALS model mice before the onset of ALS, suggesting that free radical injury and neurovascular unit damage may be closely related with ALS pathophysiology [63].

Edaravone is a drug that efficiently scavenges hydroxyl radicals [64,65]. As a phase 2 clinical study, 60 mg/day edaravone administration was performed over a 24-week period. In this clinical trial, therapeutic effects were detected as better respiratory function and suppressed expression of 3-nitrotyrosine, a marker of oxidative stress in the spinal fluid. On the basis of this result, the first phase 3 clinical trial conducted in Japan from April 2006 in ALS patients showed the effectiveness of edaravone (60 mg/day) administration, particularly on finger muscle strength, compared with the placebo group. The primary endpoint of a clinical score (the ALS functional rating scale-revised), however, was not statistically significant between the edaravone and placebo groups. For this reason, a second phase 3 clinical trial was conducted in 2012. Progression was clearly inhibited in the second clinical trial, and thus, edaravone was approved as a new anti-ALS drug [64,65]. Despite nutritional care with a gastrostomy and/or ventilatory support, which are fundamental for ALS patients, the therapeutic benefit of these treatments remains greatly limited, however, and thus, novel therapeutic strategies for ALS are in high demand. 

We recently studied the therapeutic potential of human Muse cells in transgenic mice with the G93A human SOD1 mutation, a mouse model of ALS [36]. Importantly, intravenous injection of Muse cells into the tail vein had a better homing effect to the cervical and lumbar spinal cord than intrathecal administration, indicating that intravenous injection is the best route for Muse cell administration (Figure 8B). In vivo dynamics of intravenously injected Nano-lantern-labeled cells demonstrated that the majority of human MSCs were trapped in the lungs, and a small number of them accumulated to the femur, but there were no signals in the spinal cord. In contrast, human Muse cells homed to the spinal cord, along with the lungs and femur (Figure 8C). 

The therapeutic effect of intravenous injection of human Muse cells and MSCs in the mouse ALS model revealed a significant clinical benefit in multiple behavioral tests, including the rotarod and hanging-wire tests; improved lower limb muscle strength; supported motor neuron survival; and suppressed myofiber atrophy in the Muse group, compared with the vehicle and MSC groups (Figure 8D). Homed Muse cells were suggested to mainly differentiate into glial fibrillary acidic protein-positive astrocytes in the spinal cord. The number of surviving motor neurons, as well as the number of innervated synapses and myofiber size in the tibialis anterior muscle, were significantly higher in the Muse group, compared with the vehicle and MSC groups. 

Thus, Muse cells may be a promising cell resource for the treatment of ALS patients [36].

### 4.2. Stroke

Stroke, defined as the sudden onset of neurologic deficits, triggered by cerebrovascular complications, is the second most common cause of death around the world and a major cause of disability. Among several types of stroke, two models, transient middle cerebral artery occlusion to mimic large brain infarction and lacunar infarction, in which a small subcortical infarct is induced in the corticospinal tract to produce pure motor paresis, were used for preclinical studies of Muse cells [34,38,66]. 

In subacute- and chronic-phase lacunar infarction model mice that received intravenous injection of human bone marrow-Muse cells or human Muse cell-based product CL2020, selective homing of Muse cells to the infarct region at 1 day, spontaneous differentiation of the Muse cells into NeuN(+)-, MAP-2(+)-neuronal cells, and GST-pi (+)-oligodendrocytes, and their residence in the infarcted tissue for up to 4 months, were observed with statistically meaningful recovery demonstrated in the cylinder test in a dose-dependent manner [66]. 

**Figure 9:**
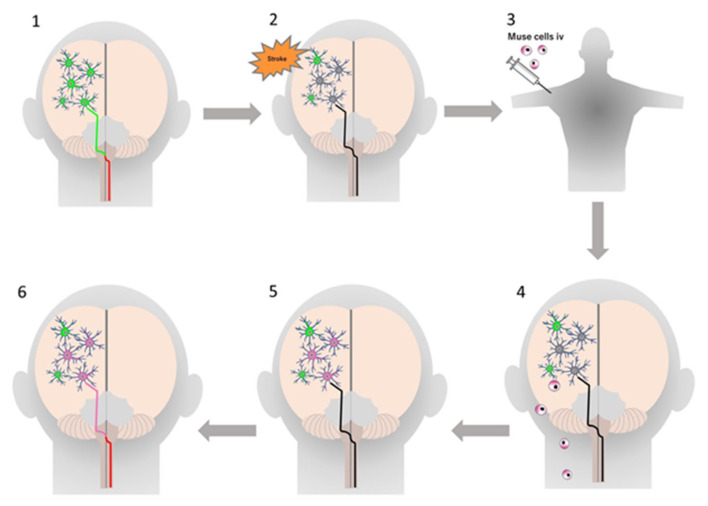
Strategy of Muse cell treatment in stroke. Muse cells selectively home to the post-infarct area in the brain after intravenous injection and spontaneously differentiate into neuronal and glial cells. Differentiated neuronal cells extend neurites that are incorporated into the pyramidal tract, including pyramidal decussation.

In an acute phase rat transient middle cerebral artery occlusion model, human dermal-Muse cells specifically homed to the peri-infarct area; spontaneously expressed neuro-progenitor markers NeuroD, Mash1, and doublecortin; and extended neurites at 3 days. At 7 days, they expressed the mature neuronal markers MAP-2 and NeuN, and the extended neurites connected with each other. Thus, commitment of Muse cells to a neuronal lineage was initiated soon after homing to the infarcted tissue and the differentiation process was swift [38]. At 3 months, the Muse cells were incorporated into the tissue as neuronal cells (~60%) and oligodendrocytes (~20%) [38]. Impressively, anterograde and retrograde labeling experiments showed that neuronal cells that differentiated from Muse cells formed new synapses connecting with motor cortex neurons, and their neurites were incorporated into the pyramidal tract and even the pyramidal decussation to reconstruct the motor circuit [34,38]. The Muse cell-derived neurites were positive for glutamatergic neuronal markers (Figure 9) [38]. Muse cells also reconstructed the sensory circuit. Human Muse cells integrated into the ipsilateral sensory cortex at 3 months and expressed synaptophysin at synapses. Furthermore, recovery of the sensory system in the Muse group was confirmed by an electrophysiologic approach, sensory evoked potentials, in that the amplitude of the sensory evoked potentials was significantly higher than that in the vehicle-treated animals [38]. 

In contrast to the Muse group, there was no significant difference between the MSC/non-Muse MSC and vehicle groups in the functional assessment, and neither MSCs nor non-Muse MSCs survived in the infarcted brain tissue as neuronal or glial cells at 3~4 months [34,38,66]. 

### 4.3. Perinatal Hypoxic Ischemic Encephalopathy

Seven-day-old rats that underwent ligation of the left carotid artery and were then exposed to 8% oxygen for 60 min received an intravenous injection of human Muse and non-Muse MSCs without immunosuppressant 72 h later [39]. Muse cells were confirmed to selectively distribute to the injured brain at 2 and 4 weeks and expressed neuronal (NeuN and MAP-2) and oligodendrocyte (GST-pi) markers at 6 months. In contrast, non-Muse MSCs became undetectable in the body within 4 weeks, and, thus, their neural differentiation was not detected. Particularly remarkable in this study was that magnetic resonance spectroscopy performed 2 days after the injection revealed a substantial reduction of excess brain glutamate metabolites in the ipsilateral brain of the Muse group. Furthermore, [^18^F]-PBR111 positron emission tomography imaging at 2 days demonstrated a significantly lower standardized uptake of [^18^F]-PBR111, an indicator of microglial activity, in the Muse group. The Muse cell-treated group also exhibited significant improvements in motor and cognitive functions for up to 5 months, compared with the vehicle and non-Muse MSC groups. In addition to the replacement of damaged neural cells by spontaneous differentiation and functional recovery, as seen in stroke models, this study newly demonstrated that Muse cells have the ability to modulate glutamate metabolism and reduce microglial activation in the infarcted brain tissue [39].

### 4.4. Intracerebral Hemorrhage

The efficiency of human Muse cells was examined in an immunodeficient mouse intracerebral hemorrhage model in which 70 µL of cardiac blood was stereotactically injected into the left putamen [40]. Five days later, Muse cells and non-Muse MSCs were injected into the intracerebral hemorrhage cavity. Motor function was tested using the Morris Water maze, and cable walking tests showed statistically significant improvement (*p* < 0.05) in the Muse group and differentiation of surviving Muse cells around the hematoma cavity into neuronal cells (NeuN (~57%) and MAP-2 (~41.6%)). On the other hand, a very small number of non-Muse MSCs survived in the tissue [40]. 

### 4.5. Shiga Toxin-Producing E. coli-Associated Acute Encephalopathy

Muse cells not only replace damaged neural cells by spontaneous differentiation but also exert anti-inflammatory, anti-apoptotic, and trophic effects and rescue STEC-associated acute encephalopathy [37]. NOD-SCID mice orally inoculated with STEC and treated 48 h later with intravenous injection of human Muse cells exhibited 100% survival and no severe after-effects of infection, while all of the vehicle-group animals died within several days due to brain edema, apoptosis of neural cells, and gliosis. Homed Muse cells spontaneously differentiated into neuronal cells, and suppressed apoptosis and gliosis. Suppression of granulocyte-colony-stimulating factor expression by RNA interference abolished the beneficial effects of Muse cells, leading to a 40% death rate and significant body weight loss. This finding suggested that granulocyte-colony-stimulating factor plays a key role in the beneficial effects of Muse cells in infection-associated encephalitis [37]. 

## 5. Clinical Trials and Future Perspectives

The cells used in preclinical research cannot be used for medical care, because the production of cells used in research typically does not meet the Good Manufacturing Practice principles, and they are not xeno-free, that is, they do not contain material from species other than human origin. A clinical-grade Muse cell product called CL2020 is currently in production by Life Science Institute Inc, a group company of the Mitsubishi Chemical Holdings Corporation (https://www.lsii.co.jp/en/; accessed on 19 April 2021). The therapeutic effects and safety of CL2020 were recently demonstrated in mouse models of epidermolysis bullosa and lacunar stroke [33,66].

Based on the safety and efficacy demonstrated in preclinical studies, six clinical trials using intravenous infusion of donor-CL2020 are currently in progress. The targets are acute myocardial infarction (started in February 2018), ischemic stroke (September 2018), epidermolysis bullosa (December 2018), spinal cord injuries (July 2019), neonatal hypoxic-ischemic encephalopathy (January 2020), and ALS (January 2021).

A first-in-human trial of Muse cells was performed in three patients with acute myocardial infarction. In this trial, 1.5 × 10^7^ clinical-grade Muse cells CL2020 (2.1 ± 0.1 × 10^5^ cells/kg) were intravenously infused at 4.1 ± 1.0 days after the onset of the infarction. One adverse effect (an increase in blood creatine phosphokinase) was observed in one of the three patients, but it was determined that this was not related to the CL2020 administration. The function of the heart, indicated by the left ventricular ejection fraction and wall motion index score, significantly improved from ~40% to ~52% after CL2020 treatment [10]. This first-in-human trial primarily suggested the safety and efficiency of Muse cells for the treatment of human diseases. However, it should be noted that this study is an open-labeled experiment, and more detailed careful analysis is required. Currently, a phase 2–3 randomized, double-blinded study is in progress at multiple facilities. 

A phase 1/2 open-label study for adult epidermolysis bullosa was also recently published. Five patients received a single injection of CL2020, and the ulcer size was significantly reduced for up to 3 months with statistical significance [11]. 

MSCs are currently widely applied in clinical studies. Nevertheless, MSCs give rise to confined cell types, chondrocytic, osteocytic, or adipocytic lineages [67], and it was recently demonstrated that the therapeutic effects of MSCs are largely explained by trophic effects, rather than cell replacement, unlike Muse cells [68]. By contrast, Muse cells possess spontaneous triploblastic differentiation ability in vivo and can specifically home to damaged tissue(s) via the S1P–S1PR2 axis. Thus, after simple intravenous administration, Muse cells can home to the site of damage and repair the damaged tissue by spontaneous, non-fusion-based differentiation [2]. Therefore, Muse cell-based therapies do not need a surgical operation to implant the cells and are easily accessible. In addition, Muse cells possess stronger DNA repair ability than MSCs [29]. The stronger DNA repair ability of Muse cells suggests a lower risk of tumorigenesis and mutation. Furthermore, because Muse cells are immune-privileged, no HLA matching or immunosuppressant treatment is needed for Muse cell-based therapies [6].

The pluripotency of Muse cells, combined with their strong reparative function and immunomodulatory properties, indicates that Muse cells may be a more realistic choice than MSCs for next-generation cell therapy. 

## Data Availability

Not applicable.

## References

[1] Kuroda Y., Kitada M., Wakao S., Nishikawa K., Tanimura Y., Makinoshima H., Goda M., Akashi H., Inutsuka A., Niwa A. (2010). Unique multipotent cells in adult human mesenchymal cell populations. Proc. Natl. Acad. Sci. USA.

[2] Kushida Y., Wakao S., Dezawa M. (2018). Muse Cells Are Endogenous Reparative Stem Cells. Adv. Exp. Med. Biol..

[3] Tanaka T., Nishigaki K., Minatoguchi S., Nawa T., Yamada Y., Kanamori H., Mikami A., Ushikoshi H., Kawasaki M., Dezawa M. (2018). Mobilized Muse Cells After Acute Myocardial Infarction Predict Cardiac Function and Remodeling in the Chronic Phase. Circ. J..

[4] Sato T., Wakao S., Kushida Y., Tatsumi K., Kitada M., Abe T., Niizuma K., Tominaga T., Kushimoto S., Dezawa M. (2020). A Novel Type of Stem Cells Double-Positive for SSEA-3 and CD45 in Human Peripheral Blood. Cell Transplant..

[5] Weigert A., Olesch C., Brüne B. (2019). Sphingosine-1-Phosphate and Macrophage Biology—How the Sphinx Tames the Big Eater. Front. Immunol..

[6] Yamada Y., Wakao S., Kushida Y., Minatoguchi S., Mikami A., Higashi K., Baba S., Shigemoto T., Kuroda Y., Kanamori H. (2018). S1P–S1PR2 Axis Mediates Homing of Muse Cells Into Damaged Heart for Long-Lasting Tissue Repair and Functional Recovery After Acute Myocardial Infarction. Circ. Res..

[7] Hori E., Hayakawa Y., Hayashi T., Hori S., Okamoto S., Shibata T., Kubo M., Horie Y., Sasahara M., Kuroda S. (2016). Mobilization of Pluripotent Multilineage-Differentiating Stress-Enduring Cells in Ischemic Stroke. J. Stroke Cerebrovasc. Dis..

[8] Dezawa M. (2016). Muse Cells Provide the Pluripotency of Mesenchymal Stem Cells: Direct Contribution of Muse Cells to Tissue Regeneration. Cell Transplant..

[9] Minatoguchi S., Mikami A., Tanaka T., Minatoguchi S., Yamada Y. (2018). Acute Myocardial Infarction, Cardioprotection, and Muse Cells. Adv. Exp. Med. Biol..

[10] Noda T., Nishigaki K., Minatoguchi S. (2020). Safety and Efficacy of Human Muse Cell-Based Product for Acute Myocardial Infarction in a First-in-Human Trial. Circ. J..

[11] Fujita Y., Nohara T., Takashima S., Natsuga K., Adachi M., Yoshida K., Shinkuma S., Takeichi T., Nakamura H., Wada O. (2021). Intravenous allogeneic multilineage-differentiating stress-enduring cells in adults with dystrophic epidermolysis bullosa: A phase 1/2 open-label study. J. Eur. Acad. Dermatol. Venereol..

[12] Shevinsky L.H., Knowles B.B., Damjanov I., Solter D. (1982). Monoclonal antibody to murine embryos defines a stage-specific embryonic antigen expressed on mouse embryos and human teratocarcinoma cells. Cell.

[13] Kannagi R., Cochran N.A., Ishigami F., Hakomori S., Andrews P.W., Knowles B.B., Solter D. (1983). Stage-specific em-bryonic antigens (SSEA-3 and -4) are epitopes of a unique globo-series ganglioside isolated from human teratocarcino-ma cells. EMBO J..

[14] Yang Z., Liu J., Liu H., Qiu M., Liu Q., Zheng L., Pang M., Quan F., Zhang Y. (2013). Isolation and Characterization of SSEA3+ Stem Cells Derived from Goat Skin Fibroblasts. Cell. Reprogr..

[15] Nitobe Y., Nagaoki T., Kumagai G., Sasaki A., Liu X., Fujita T., Fukutoku T., Wada K., Tanaka T., Kudo H. (2019). Neurotrophic Factor Secretion and Neural Differentiation Potential of Multilineage-differentiating Stress-enduring (Muse) Cells Derived from Mouse Adipose Tissue. Cell Transplant..

[16] Iseki M., Mizuma M., Wakao S., Kushida Y., Kudo K., Fukase M., Ishida M., Ono T., Shimura M., Ise I. (2021). The evaluation of the safety and efficacy of intravenously administered allogeneic multilineage-differentiating stress-enduring cells in a swine hepatectomy model. Surg. Today.

[17] Sun D., Yang L., Cao H., Shen Z., Song H. (2020). Study of the protective effect on damaged intestinal epithelial cells of rat multilineage-differentiating stress-enduring (Muse) cells. Cell Biol. Int..

[18] Wakao S., Kushida Y., Dezawa M. (2019). Correction to: Basic Characteristics of Muse Cells. Muse Cells.

[19] Leng Z., Sun D., Huang Z., Tadmori I., Chiang N., Kethidi N., Sabra A., Kushida Y., Fu Y.-S., Dezawa M. (2019). Quantitative Analysis of SSEA3+ Cells from Human Umbilical Cord after Magnetic Sorting. Cell Transplant..

[20] Rompolas P., Greco V. (2014). Stem cell dynamics in the hair follicle niche. Semin. Cell Dev. Biol..

[21] Boulais P.E., Frenette P.S. (2015). Making sense of hematopoietic stem cell niches. Blood.

[22] Wakao S., Kitada M., Kuroda Y., Shigemoto T., Matsuse D., Akashi H., Tanimura Y., Tsuchiyama K., Kikuchi T., Goda M. (2011). Multilineage-differentiating stress-enduring (Muse) cells are a primary source of induced pluripotent stem cells in human fibroblasts. Proc. Natl. Acad. Sci. USA.

[23] Tsuchiyama K., Wakao S., Kuroda Y., Ogura F., Nojima M., Sawaya N., Yamasaki K., Aiba S., Dezawa M. (2013). Functional Melanocytes Are Readily Reprogrammable from Multilineage-Differentiating Stress-Enduring (Muse) Cells, Distinct Stem Cells in Human Fibroblasts. J. Investig. Dermatol..

[24] Amin M., Kushida Y., Wakao S., Kitada M., Tatsumi K., Dezawa M. (2018). Cardiotrophic Growth Factor–Driven Induction of Human Muse Cells into Cardiomyocyte-Like Phenotype. Cell Transplant..

[25] Kuroda Y., Wakao S., Kitada M., Murakami T., Nojima M., Dezawa M. (2013). Isolation, culture and evaluation of multilineage-differentiating stress-enduring (Muse) cells. Nat. Protoc..

[26] Ogura F., Wakao S., Kuroda Y., Tsuchiyama K., Bagheri M., Heneidi S., Chazenbalk G., Aiba S., Dezawa M. (2014). Human Adipose Tissue Possesses a Unique Population of Pluripotent Stem Cells with Nontumorigenic and Low Telomerase Activities: Potential Implications in Regenerative Medicine. Stem Cells Dev..

[27] Iseki M., Kushida Y., Wakao S., Akimoto T., Mizuma M., Motoi F., Asada R., Shimizu S., Unno M., Chazenbalk G. (2017). Human Muse Cells, Nontumorigenic Phiripotent-Like Stem Cells, Have Liver Regeneration Capacity through Specific Homing and Cell Replacement in a Mouse Model of Liver Fibrosis. Cell Transplant..

[28] Alessio N., Özcan S., Tatsumi K., Murat A., Peluso G., Dezawa M., Galderisi U. (2017). The secretome of MUSE cells contains factors that may play a role in regulation of stemness, apoptosis and immunomodulation. Cell Cycle.

[29] Alessio N., Squillaro T., Özcan S., Di Bernardo G., Venditti M., Melone M., Peluso G., Galderisi U. (2018). Stress and stem cells: Adult Muse cells tolerate extensive genotoxic stimuli better than mesenchymal stromal cells. Oncotarget.

[30] Gimeno M.L., Fuertes F., Tabarrozzi A.E.B., Attorressi A.I., Cucchiani R., Corrales L., Oliveira T.C., Sogayar M.C., Labriola L., Dewey R.A. (2016). Pluripotent Nontumorigenic Adipose Tissue-Derived Muse Cells have Immunomodulatory Capacity Mediated by Transforming Growth Factor-β1. Stem Cells Transl. Med..

[31] Milstien S., Spiegel S. (2004). Generation and metabolism of bioactive sphingosine-1-phosphate. J. Cell. Biochem..

[32] De Becker A., Van Riet I. (2016). Homing and migration of mesenchymal stromal cells: How to improve the efficacy of cell therapy?. World J. Stem Cells.

[33] Fujita Y., Komatsu M., Lee S.E., Kushida Y., Nakayama-Nishimura C., Matsumura W., Takashima S., Shinkuma S., Nomura T., Masutomi N. (2021). Intravenous Injection of Muse Cells as a Potential Therapeutic Approach for Epidermolysis Bullosa. J. Investig. Dermatol..

[34] Uchida H., Niizuma K., Kushida Y., Wakao S., Tominaga T., Borlongan C.V., Dezawa M. (2017). Human Muse Cells Reconstruct Neuronal Circuitry in Subacute Lacunar Stroke Model. Stroke.

[35] Uchida N., Kushida Y., Kitada M., Wakao S., Kumagai N., Kuroda Y., Kondo Y., Hirohara Y., Kure S., Chazenbalk G. (2017). Beneficial Effects of Systemically Administered Human Muse Cells in Adriamycin Nephropathy. J. Am. Soc. Nephrol..

[36] Yamashita T., Kushida Y., Wakao S., Tadokoro K., Nomura E., Omote Y., Takemoto M., Hishikawa N., Ohta Y., Dezawa M. (2020). Therapeutic benefit of Muse cells in a mouse model of amyotrophic lateral sclerosis. Sci. Rep..

[37] Ozuru R., Wakao S., Tsuji T., Ohara N., Matsuba T., Amuran M.Y., Isobe J., Iino M., Nishida N., Matsumoto S. (2020). Rescue from Stx2-Producing *E. coli*-Associated Encephalopathy by Intravenous Injection of Muse Cells in NOD-SCID Mice. Mol. Ther..

[38] Uchida H., Morita T., Niizuma K., Kushida Y., Kuroda Y., Wakao S., Sakata H., Matsuzaka Y., Mushiake H., Tominaga T. (2016). Transplantation of Unique Subpopulation of Fibroblasts, Muse Cells, Ameliorates Experimental Stroke Possibly via Robust Neuronal Differentiation. Stem Cells.

[39] Suzuki T., Sato Y., Kushida Y., Tsuji M., Wakao S., Ueda K., Imai K., Iitani Y., Shimizu S., Hida H. (2020). Intra-venously delivered multilineage-differentiating stress enduring cells dampen excessive glutamate metabolism and mi-croglial activation in experimental perinatal hypoxic ischemic encephalopathy. J. Cereb. Blood Flow.

[40] Shimamura N., Kakuta K., Wang L., Naraoka M., Uchida H., Wakao S., Dezawa M., Ohkuma H. (2017). Neuro-regeneration therapy using human Muse cells is highly effective in a mouse intracerebral hemorrhage model. Exp. Brain Res..

[41] Katagiri H., Kushida Y., Nojima M., Kuroda Y., Wakao S., Ishida K., Endo F., Kume K., Takahara T., Nitta H. (2016). A Distinct Subpopulation of Bone Marrow Mesenchymal Stem Cells, Muse Cells, Directly Commit to the Replacement of Liver Components. Arab. Archaeol. Epigr..

[42] Hosoyama K., Wakao S., Kushida Y., Ogura F., Maeda K., Adachi O., Kawamoto S., Dezawa M., Saiki Y. (2018). Intravenously injected human multilineage-differentiating stress-enduring cells selectively engraft into mouse aortic aneurysms and attenuate dilatation by differentiating into multiple cell types. J. Thorac. Cardiovasc. Surg..

[43] Ankrum J.A., Ong J.F., Karp J.M. (2014). Mesenchymal stem cells: Immune evasive, not immune privileged. Nat. Biotechnol..

[44] Zhao L., Chen S., Yang P., Cao H., Li L. (2019). The role of mesenchymal stem cells in hematopoietic stem cell transplantation: Prevention and treatment of graft-versus-host disease. Stem Cell Res. Ther..

[45] Najima Y., Ohashi K. (2017). Mesenchymal Stem Cells: The First Approved Stem Cell Drug in Japan. J. Hematop. Cell Transplant..

[46] Loustau M., Anna F., Dréan R., LeComte M., Langlade-Demoyen P., Caumartin J. (2020). HLA-G Neo-Expression on Tumors. Front. Immunol..

[47] Yabuki H., Wakao S., Kushida Y., Dezawa M., Okada Y. (2018). Human Multilineage-differentiating Stress-Enduring Cells Exert Pleiotropic Effects to Ameliorate Acute Lung Ischemia–Reperfusion Injury in a Rat Model. Cell Transplant..

[48] Shono Y., Kushida Y., Wakao S., Kuroda Y., Unno M., Kamei T., Miyagi S., Dezawa M. (2020). Protection of liver sinusoids by intravenous administration of human Muse cells in a rat extra-small partial liver transplantation model. Arab. Archaeol. Epigr..

[49] Surgucheva I., Chidambaram K., Willoughby D.A., Surguchov A. (2010). Matrix metalloproteinase 9 expression: New regulatory elements. J. Ocul. Biol. Dis. Inform..

[50] Ratajczak M.Z., Shin D.-M., Liu R., Mierzejewska K., Ratajczak J., Kucia M., Zuba-Surma E.K. (2012). Very small embryonic/epiblast-like stem cells (VSELs) and their potential role in aging and organ rejuvenation—An update and comparison to other primitive small stem cells isolated from adult tissues. Aging.

[51] Jiang Y., Vaessen B., Lenvik T., Blackstad M., Reyes M., Verfaillie C.M. (2002). Multipotent progenitor cells can be isolated from postnatal murine bone marrow, muscle, and brain. Exp. Hematol..

[52] Nichols J., Smith A. (2009). Naive and Primed Pluripotent States. Cell Stem Cell.

[53] Ying Q.-L., Nichols J., Chambers I., Smith A. (2003). BMP Induction of Id Proteins Suppresses Differentiation and Sustains Embryonic Stem Cell Self-Renewal in Collaboration with STAT3. Cell.

[54] Tesar P.J., Chenoweth J.G., Brook F.A., Davies T.J., Evans E.P., Mack D.L., Gardner R.L., McKay R.D.G. (2007). New cell lines from mouse epiblast share defining features with human embryonic stem cells. Nat. Cell Biol..

[55] Masrori P., Van Damme P. (2020). Amyotrophic lateral sclerosis: A clinical review. Eur. J. Neurol..

[56] Ogasawara M., Matsubara Y., Narisawa K., Aoki M., Nakamura S., Itoyama Y., Abe K. (1993). Mild ALS in Japan associated with novel SOD mutation. Nat. Genet..

[57] Gurney M.E., Pu H., Chiu A.Y., Dal Canto M.C., Polchow C.Y., Alexander D.D., Caliendo J., Hentati A., Kwon Y.W., Deng H.X. (1994). Motor neuron degeneration in mice that express a human Cu, Zn superoxide dismutase mutation. Science.

[58] Arai T., Hasegawa M., Akiyama H., Ikeda K., Nonaka T., Mori H., Mann D., Tsuchiya K., Yoshida M., Hashizume Y. (2006). TDP-43 is a component of ubiquitin-positive tau-negative inclusions in frontotemporal lobar degeneration and amyotrophic lateral sclerosis. Biochem. Biophys. Res. Commun..

[59] DeJesus-Hernandez M., Mackenzie I.R., Boeve B.F., Boxer A.L., Baker M., Rutherford N.J., Nicholson A.M., Finch N.A., Flynn H., Adamson J. (2011). Expanded GGGGCC Hexanucleotide Repeat in Noncoding Region of C9ORF72 Causes Chromosome 9p-Linked FTD and ALS. Neuron.

[60] Paré B., Lehmann M., Beaudin M., Nordström U., Saikali S., Julien J.-P., Gilthorpe J.D., Marklund S.L., Cashman N.R., Andersen P.M. (2018). Misfolded SOD1 pathology in sporadic Amyotrophic Lateral Sclerosis. Sci. Rep..

[61] Ferrante R.J., Browne S.E., Shinobu L.A., Bowling A.C., Baik M.J., MacGarvey U., Kowall N.W., Brown R.H., Beal M.F. (2002). Evidence of Increased Oxidative Damage in Both Sporadic and Familial Amyotrophic Lateral Sclerosis. J. Neurochem..

[62] Warita H., Hayashi T., Murakami T., Manabe Y., Abe K. (2001). Oxidative damage to mitochondrial DNA in spinal motoneurons of transgenic ALS mice. Mol. Brain Res..

[63] Miyazaki K., Ohta Y., Nagai M., Morimoto N., Kurata T., Takehisa Y., Ikeda Y., Matsuura T., Abe K. (2011). Disruption of neurovascular unit prior to motor neuron degeneration in amyotrophic lateral sclerosis. J. Neurosci. Res..

[64] Abe K., Itoyama Y., Sobue G., Tsuji S., Aoki M., Doyu M., Hamada C., Kondo K., Yoneoka T., Akimoto M. (2014). Confirmatory double-blind, parallel-group, placebo-controlled study of efficacy and safety of edaravone (MCI-186) in amyotrophic lateral sclerosis patients. Amyotroph. Lateral Scler. Front. Degener..

[65] Abe K., Aoki M., Tsuji S., Itoyama Y., Sobue G., Togo M., Hamada C., Tanaka M., Akimoto M., Nakamura K. (2017). Safety and efficacy of edaravone in well defined patients with amyotrophic lateral sclerosis: A randomised, double-blind, placebo-controlled trial. Lancet Neurol..

[66] Abe T., Aburakawa D., Niizuma K., Iwabuchi N., Kajitani T., Wakao S., Kushida Y., Dezawa M., Borlongan C.V., Tominaga T. (2020). Intravenously Transplanted Human Multilineage-Differentiating Stress-Enduring Cells Afford Brain Repair in a Mouse Lacunar Stroke Model. Stroke.

[67] Pittenger M.F., Mackay A.M., Beck S.C., Jaiswal R.K., Douglas R., Mosca J.D., Moorman M.A., Simonetti D.W., Craig S., Marshak D.R. (1999). Multilineage Potential of Adult Human Mesenchymal Stem Cells. Science.

[68] Fu Y., Karbaat L., Wu L., Leijten J., Both S.K., Karperien M. (2017). Trophic Effects of Mesenchymal Stem Cells in Tissue Regeneration. Tissue Eng. Part B Rev..

